# Remarks upon the Morbid Anatomy of the Brain in Insanity

**Published:** 1851-04-01

**Authors:** Holmes Coote

**Affiliations:** ENGLAND, DEMONSTRATOR OF ANATOMY AT SAINT BARTHOLOMEW'S HOSPITAL


### REMARKS UPON THE MORBID ANATOMY OF THE BRAIN IN INSANITY.

BY HOLMES COOTE, F. R. COLL. SURG. ENGLAND, DEMONSTRATOR OF ANATOMY AT SAINT BARTHOLOMEW'S HOSPITAL.

In the examination of the bodies of those who die insane, we find the morbid appearances most frequent and most strongly marked upon the surface of the cerebral hemispheres; in those structures where the bloodvessels arrange themselves in a closely woven net-work before penetrating the cerebral substance. There is no contrast more marked than that between the thin, delicate, and transparent membranes covering the perfectly healthy brain, and the thickened and opaque arachnoid, and the infiltrated pia mater, which invest the convolutions of the hemispheres of one, whose cerebral circulation has long been disturbed and excited. But then, considering that no such morbid appearances are found in the bodies of some, who, as in the instance of puerperal maniacs, die sometimes shortly after the manifestation of the malady, we must, I think, conclude that such changes of structure are rather to be regarded as the effect of excited circulation, dependent upon, or co-existent with insanity, than as the cause of the disease itself. For an account of the conditions of the membranes of the brain, I refer to a preceding report.

The lateral and the third ventricles lined by a ciliated membrane different from the arachnoid, and containing upon their floor the choroid plexuses, which are derived from the vessels of the pia mater, do not communicate in the adult brain with the general serous cavity by the fissure of Bichât. The communication which existed in fœtal life is closed, hence we must not expect to find the morbid appearances of the surface repeated in the interior of the brain. Such an occurrence may be remarked in instances of acute inflammation of the pia mater, but not as a rule in the more ordinary and chronic forms of meningitis. In the following case, pus was found both on the surface of the convolutions and in the ventricles.

Case I.—Elizabeth E., examined March 22, 1849. Over the entire surface of both cerebrum and cerebellum, the pia mater was infiltrated by yellowish green purulent fluid, which accurately followed the convolutions of the brain. . . . . . . The ventricles contained about three ounces of turbid yellow purulent fluid; the same morbid appearances were noticed upon the surface of the spinal chord.

The following is an instance of thickening and opacity of the arachnoid over the whole cerebral surface; the lining membrane of the ventricles retaining its natural transparency: the slight increase of limpid fluid in the interior being derived from the vessels of the pia mater.

Case II.—William S., examined January 8, 1845. The bloodvessels of the cranium, the membranes and the brain were very turgid, the latter particularly so, the medullary substance being full of bloody points, wherever it was cut into. The arachnoid was thickened and opaque, especially along the sides of the great fissure of the cerebrum. It was slightly milky over the whole hemispheres. There was considerable infiltration of the pia mater, and some increase of fluid in the ventricles. The structure and firmness of the brain were natural.

There were some partial adhesions of both lungs, of old standing; strong and close adhesions of the pericardium; the right kidney shrunk to one-third of its natural size; slight diminution of the left.

Were all necroscopical researches recorded after the fashion of Dr. Greding, we might be disposed to think with Pinel, that it is hopeless to expect, by such observations, to elucidate the pathology of mental derangements. When we read that out of a hundred cases, the lateral ventricles were very full of serum in twenty-nine cases; ready to burst in twenty-three; astonishingly distended in ten; the third ventricle quite full in fifty-seven maniacs, and in sixteen of twenty-four melancholics, we are led to ask, was the communication between the ventricles closed in those cases that distention of only one cavity was observed? If such were the case, it is a phenomenon worth recording, and the particulars should be carefully stated; for, as a rule, we observe that with the distention of the lateral ventricles, the foramina of Monro leading to the third ventricle have been enlarged by the separation of the optic thalami from the fornix. We find in the same report, "Fourth ventricle *ready to burst* in eighty out of a hundred maniacs; quite empty in only three; completely distended in every one of twenty-four melancholics. Now, the fourth ventricle is the space between the under surface of the cerebellum and the upper surface of the spinal chord. It communicates anteriorly with the third ventricle by a narrow canal, and posteriorly it is shut up by the arachnoid membrane and pia mater, which are loosely reflected from the chord. What structure was ready to burst? As the membranes would readily separate from the parts which they cover to allow of any amount of effusion; and as it is clear the anterior boundary, in which the *iter e tertio ad quartum ventriculum* opens, is incapable of giving way, it follows that the danger of bursting must refer to some part of the cerebellum *or* medulla oblongata. Such an observation involves a *primâ facie* absurdity. Again, in the three instances in which the ventricle is pronounced empty, it is but reasonable to infer that the fluid, small in quantity, as is usually the case, had run out during the examination. In speaking of distention of the lateral ventricles, it is always implied that the walls of the third and of the fourth ventricles have been exposed to a similar change; I never have seen a case in which fulness of the third or fourth ventricle could be recorded as a special observation. The fluid in the ventricles, consisting of water with a small proportion of albumen, is not uncommonly poured forth in large quantity, when, in combination with the effusion of fluid on the surface of the brain, it produces an amount of pressure sufficient to cause death. Instances of serous apoplexy are not very uncommon in Bethlehem Hospital; sanguineous apoplexy is rare.

In serous apoplexy, the arachnoid sac usually contains no fluid: there is a variable amount of fluid in the pia mater; the convolutions of the brain are flat and compressed, and the whole organ seems too large for the cavity of the skull.

The following case illustrates the appearances after death:—

Case III.—Maria W. S., aged 36, admitted into the curable establishment March 31, 1846; died Feb. 9, 1850. The skull-cap was heavy, and the cancellous texture was obliterated; the inner surface was rough, presenting prominences which, projecting into the cranial cavity, pressed against the dura mater, which was rendered thin and transparent, and against the upper and front part of the cerebral hemispheres, which were flattened. Upon the removal of the skull-cap, the brain bulged over the sawn edges of the bone, as if liberated from pressure. The cerebral substance was white and soft; the ventricles were enormously distended, and contained full four ounces of clear watery fluid. There was a large quantity of fluid at the base of the skull. There were traces of old tuberculous disease in the chest, and there were numerous tuberculous ulcers along the course of the intestinal canal.

In the greater number of the cases, the amount of fluid in the ventricles varies from an ounce and a half to three ounces. The normal quantity may, perhaps, be estimated at two or three drachms. In a report furnished by Dr. Webster to the Transactions of the Medical and Chirurgical Society, it is stated that, out of seventy-two examinations conducted by Mr. Lawrence, in Bethlehem Hospital, an increase of fluid in the ventricles was noticed forty-one times.

And yet this serous effusion cannot be regarded as essentially connected with insanity; for the same morbid appearances, to an equal extent, are seen in those who retain their faculties to the last, and describe, in graphic language, the sensations which they experience from this internal pressure.

Case IV.—A beadle in a public institution, who, after living an intemperate life, became the subject of gout and disease of the heart; suffered, for three or four years before his death, extreme distress from weight of the head, pain about the temples, and dizziness upon making any exertion. Upon several occasions, the sudden sound of a street-door knock, or a sharp ring of a bell, would cause him to fall down senseless. He walked about with the air of one labouring under some affection of the brain, the head being kept motionless, turning with the whole trunk; as if he feared even to rotate his face from side to side. Although blood was frequently and freely abstracted upon any exacerbation of the symptoms, the relief thus afforded was but partial and temporary. One morning, after having passed two or three days of more than usual distress, but with his intellect as usual, and in the ordinary discharge of his duties, he fell from his chair senseless. The breathing soon became stertorous, the mouth was drawn to one side, and he died with the usual symptoms of an apoplectic stroke.

Upon examination of the body it was found that the dura mater adhered firmly to the skull-cap: there was rather slight thickening and opacity of the arachnoid, along the course of the superior longitudinal fissure. The pia mater was infiltrated to some extent by serum, but the cerebral convolutions were closely packed together and flattened. The ventricles contained about three ounces of limpid fluid. In the right hippo campal, or middle lobe of the brain, there was a soft dark clot of recently extravasated blood; the cerebral substance around was broken, soft, and discoloured; and the fluid on the descending corner of the right lateral ventricle (which was entire) was slightly tinged with blood.

There was hypertrophy of the left ventricle of the heart.

In sanguineous apoplexy, an accident generally connected with hypertrophy of the left ventricle of the heart, the extravasation, as in the case just related, takes place, (as far as the evidence goes from the examination of the bodies in Bethlehem Hospital,) in the cerebral substance, and not in the cavity of the ventricles. If in the immediate proximity of the ventricle, the blood stains the fluid contained in the interior of a light pink, of pale straw, or of deep reddish brown hue; the sides of the cavity may be so pressed together, and the apoplectic cell have so well defined a boundary, that a mistake as to the true situation of the extravasation may readily occur, if the examination be hastily conducted.

It has not hitherto occurred to me to witness distention of any of the ventricles with blood, in a case of pure sanguineous apoplexy, although, for a considerable time, I have made post mortem examination for the purpose of investigating the point.

The following case illustrates well the morbid appearances:—

Case V.—James A. B., aged 45, admitted into the Incurable Establishment, January 11th, 1839; died, February 18th, 1849.

The external vessels of the head were empty. After the skull-cap had been removed, the dura mater was tense; and on division of that membrane, the brain bulged a little over the edges of the bone, as if it had been previously compressed. The surfaces of the arachnoid membrane were comparatively free from moisture, and the convolutions of the cerebrum were partially flattened. In the substance of the left hemisphere there was a lacerated cavity, with broken and irregular surface, containing at least three ounces of coagulated blood. The cavity was principally in the middle lobe, but it extended into the anterior and the posterior lobes. It came close to the exterior wall of the ventricle, in which no breach of surface could be detected, although the fluid in the cavity was deeply tinged with blood, whilst that in the right ventricle was similarly tinged in a less degree. The upper surface of this cavity was on a level with the roof of the ventricle. The cavity contained a firm recent black coagulum, with thickish fluid blood of the same colour. The substance of the brain was tinged of a reddish-brown hue, to the extent of half-an-inch or three-quarters all round, the colour being deep close to the excavation; and gradually shaded off for another half-inch, the medullary substance was of a light yellow colour. The consistence of the discoloured portion did not differ much from that of healthy cerebral substance. The brain, in other respects, and the arterial trunks at the base, with their ramifications, were quite healthy.

Slight effusion of fibrine on the lower edge of the upper lobe of the right lung, and incipient consolidation of the neighbouring pulmonary substance. These changes were of very small extent. General old adhesions of the left lung, which was quite healthy in other respects.

Concentric hypertrophy of the left ventricle, of which the muscular substance was compact and firm.

The abdominal viscera were healthy.

It is affirmed by Dr. Greding, that the "Plexus choroides was seen in a perfectly healthy state in only 16 out of 216 cases which he had the opportunity of examining." That statement is completely at variance with my observations. If the brain is bloodless, the plexus choroides is pale; if the cerebral vessels, and the vessels of the pia mater are full, it may be congested and of red colour; but I cannot recal a single instance in which it could be pronounced diseased, unless, indeed, those cases be selected in which small watery cysts are found amongst the veins of which it is chiefly composed.* But these cysts are just as frequently met with in the bodies of those who die in other institutions and from other diseases; and they cannot even be said to be in any way connected with or dependent upon that morbid condition of the cerebral circulation, of which traces are so commonly met with in the cranium of the insane. The vesicular or cortical substance of the convolutions presents in some rare instances of intense congestion a light pinkish grey colour, perceptible more especially at the junction of the white and grey matter. In the cases of chronic congestion it occasionally becomes so adherent to the pia mater, that upon the separation of that membrane, portions of it remain attached to its cerebral surface, and the part of the brain, which is exposed, has a rough and broken appearance.

M. Foville asserts that, "in the most acute cases the surface of the cortical substance presents, on the removal of the membranes, a *most intense redness* approaching to that of erysipelas." It appears to me that the language here employed conveys an idea of redness so far beyond that amount which has ever been observed in this country, that it should be received with considerable hesitation. The usual grey colour of the con-

* For the mode of development of cysts from veins, I refer to a Clinical Lecture delivered by Mr. Lawrence, in St. Bartholomew's Hospital, and reported in the "Medical Times," Nov. 30, 1850.

volutions is rarely if ever materially changed; and certainly an approach to the pink colour of erysipelas would be a phenomenon.

We hear much of a morbid condition in the consistence of the brain: that it is either harder or softer than natural; and the minute changes have been described upon which such conditions depend. M. Calmeil speaks of the grey substance contiguous to the pia mater being softened, and having the consistence of the pulp of a rotten apple. "This ramollissement extends to the depth of a quarter or half a line. We conclude," adds M. Calmeil, "that the want of cohesion in the grey matter is the result of inflammation." To phlegmasia, under different circumstances, and in a different modification, he likewise ascribes the hardening of the convolutions observed in some rare instances of the disease. I trust to be pardoned remarking, that in the records of necroscopical researches there has been very great looseness in the employment of the terms "hardening and softening." Before we have any right to assume that alterations in the consistence of the brain, as commonly witnessed, unconnected with disorganization of the tissues, and not extreme in degree, should be recorded amongst the morbid appearances, there should be taken into consideration, the patient's condition during life, and the state of the blood; the age; the mode of death; the period which elapses before the post mortem examination; the time of year; the condition of the atmosphere; and the place where the body has been kept. According to the records of Bethlehem Hospital morbid hardening and softening of the cerebral substance are extremely uncommon appearances; for I presume that the superficial softening, one line in thickness, mentioned by M. Calmeil, easy to be found if sought for, would not, in the present state of knowledge upon the subject, find a ready access to our works on morbid anatomy. Mr. Lawrence has described the following instance of "hardening of the brain," in the list of cases from which the remarks contained in this report are drawn.

Cranium considerably below average adult size; bone thicker than usual, so that the skull-cap was very heavy. Cerebrum proportionately small, but cerebellum not at all below its usual dimensions. Anterior cerebral lobes particularly small, and convolutions very narrow. Cerebral substance so firm as to resemble a brain hardened by alcohol or strong acid. Lateral ventricles enlarged; each contained about two ounces of clear colourless fluid. Septum lucidum extremely thin, and torn towards its front part. Substance of the brain above the ventricle not thicker than the third of an inch.

Softening of the cerebral substance may result from more causes than one. It is stated to ensue from chronic inflammation: we more frequently meet with it as a consequence of laceration of the brain by extravasation of blood; or by a sudden effusion of serum into the ventricles. Of the former, some cases have already been related. The following is an instance of the latter:—

Case VI.—Alexander M——, examined Jan. 20, 1848. General, but not great emaciation. The external vessels of the head completely empty; those of the membranes and brain turgid. The dura mater and the cerebral hemispheres were in the closest contact; the convolutions of the latter completely flattened. The lateral ventricles greatly enlarged and distended by a slightly turbid fluid, estimated at three ounces in each cavity.

The posterior portion of the fornix, in the extent of about an inch, was irregularly torn, but not completely through, the edges presenting torn shreds floating in the fluid, and all the appearances of recent laceration. Although this might possibly have occurred in the examination, there was little doubt that it had taken place before death, as a consequence of that sudden effusion into the ventricles which had subjected the whole cranial contents to pressure, and thus caused the flattening of the convolutions, the latter appearance being as strongly marked as in sanguineous apoplexy. There was extensive tuberculous deposit in the viscera, both of the thorax and abdomen. The lungs were studded with tubercles, of which some had softened into cavities. The peritoneum presented a tuberculated appearance throughout; near to the liver there were some accumulations equal in size to walnuts, and masses of this apparently inorganic deposit caused general accretion of the opposed surfaces of the diaphragm, liver, stomach, duodenum, pancreas, and colon.

It is not in all instances possible to determine after death the cause of "softening." In the record, the doubt, if it exists, should in each case be expressed.

A young lady, aged 19, became deranged in consequence of family distresses, by which her parents were reduced from affluence to comparative poverty. She died, after having passed through a short period of silent despondency. The body was examined December 13th, 1847. The external vessels of the head were completely empty; the internal, on the contrary, with those of the brain and of its membranes, were in the highest state of congestion. The adhesion of the dura mater to the skull was particularly strong. The vessels of the dura mater, pia mater, and cerebral substance, were injected, to their minutest ramifications, with blood. A few drachms of watery fluid, slightly tinged with blood, ran out of the arachnoid cavity on cutting round the dura mater.

The pia mater was greatly infiltrated throughout; there were a few drachms of fluid in the lateral ventricles, and much fluid remained in the base of the skull after the brain had been removed.

The substance both of the cerebrum and cerebellum was particularly soft throughout. (Whether this softness was sufficient to constitute a pathological condition, might be doubted.)

There was a firm old adhesion, of limited extent, at the upper and back part of the right lung; the substance of the viscus was particularly hard for a space not exceeding the diameter of a shilling, and contained a little knot of dry, hard, whitish substance, apparently earthy. The contents of the thorax, in other respects, and those of the abdomen, were healthy.

It is not common to find the brain in just that condition which we should pronounce healthy, although such is sometimes the case. The vessels are generally preternaturally full, or so empty that the brain is described as bloodless. Softening sometimes occurs in patches on the surface of the brain, from limited extravasations of blood. The following is a good instance of the kind:—

I was requested, in the spring of 1849, by Mr. Newton, of Howland-street, to examine the body of an old lady who died deranged. She had been very violent at different periods of her disease, but for some time before death she had become bedridden, and almost paralyzed. The skull-cap was heavy, and the dura mater adhered firmly to the cranium; the arachnoid surface was lined by a thick layer of organized lymph, which formed a continuous adventitious membrane, down to the margin of the foramen magnum. There were three spots of softened and disorganized cerebral substance upon the surface of the right hemisphere. Two were the size of walnuts, one the size of a pea. The nerve-tubes were broken up, and mixed with a quantity of extravasated blood-discs, nerve-cells, granular matter, and portions of capillary vessels. There was a similar spot upon the upper surface of the middle lobe of the left hemisphere. There was effusion into the pia mater, the fluid being clear and limpid, and filling up wide spaces between the convolutions, which were narrow and shrunken.

The other viscera were not examined.

Tuberculous disease of the brain and of its membranes has been described, and preparations illustrating the former are to be found in the museums of many hospitals. It is worthy of remark, that such morbid appearances are not common amongst the insane. Indeed, I cannot recal an instance of it out of 150 cases. And this is the more remarkable, as, in many, tuberculous deposit had gone on to a very considerable extent in the viscera of the thorax and abdomen. Amongst these cases are to be found instances of tuberculosis of the peritoneum, of the pancreas, and of the stomach; both lungs may be infiltrated by tubercle, or the pleura may present an uneven but continuous tuberculated appearance, from the same cause. But in the brain and in its membranes such a change is undoubtedly rare, at least amongst that class of patients who, coming from all parts of the country, are received within the walls of Bethlehem Hospital.

Atrophy and shrinking of the cerebral convolutions is a more common appearance, especially amongst the aged. In an institution where the greater number of the patients are curable, and in whom insanity has manifested itself at some late period of life, in consequence of external causes operating upon a brain which has performed its functions healthily up to a certain point, it is uncommon to meet with instances of congenital malformation. Deficiency of the commissures, especially of the corpus callosum, is always associated with feeble intellect, as has been proved by the cases of Reil, Paget, and Mitchell Henry. Patients in whom such a malformation exists, generally remain weak-minded, but harmless, and would, if unfit to be trusted by themselves, be more properly consigned to an asylum for idiots, than to an hospital for the insane. There is no prospect of cure where the derangement is connected with arrest of the development of the brain.

The only instance of malformation noticed in the list of post-mortem examinations, which have been conducted in Bethlehem since the year 1840, is the following:—

Case 7.—Elizabeth S., aged 48, admitted on the curable establishment, December 29th, 1848; died, January 5th, 1849.

The vessels of the dura mater were full of blood; the arachnoid membrane was thickened and opake. The layers of the pia mater were separated and infiltrated by an enormous effusion of reddish brown serum. The convolutions were atrophied; the ventricles contained about three ounces of clear limpid serum.

The ventricle of the septum lucidum (the fifth ventricle) was distended by serum. It measured two inches and a half in length, and a quarter of an inch in breadth; the left wall was adherent in one spot to the anterior part of the left corpus striatum. The foramina of Monro were open, and of oval form. There was a congenital longitudinal fissure along the middle of the fornix.

The lungs presented numerous firm black spots, about the size of a split pea; there were accumulations of carbon around the minute bronchi and the air-cells. The other organs were healthy.

It would be interesting, were it possible, to ascertain the condition of the circulating fluid. The very frequent complication of organic diseases in the thoracic or abdominal viscera renders it probable that for a long period previous to death, in a great number of cases, the state of the blood is far from healthy. The pallid face, the pinched look, the cold shrunken extremities, are incompatible with the healthy circulation of well-organized blood. Examination after death may detect partial congestions, but in most cases the different viscera are pale and bloodless. Decomposition occasionally goes on with a rapidity quite unusual, surpassing that with which we are all familiar in fevers, where the skin and the subcutaneous tissues are stained along the course of the large superficial veins by the transudation of partly decomposed blood.

Case 8.—William A. B., aged 41, a patient on the curable establishment, died April 6th, 1850, the weather being fine, dry, and moderately warm. He was examined between sixty and seventy hours afterwards.

There was an incised wound, about two inches and a half in length, of green colour, and with fœtid odour, upon the forehead. After removing the skull-cap, which was of dark colour, but healthy, it was found that the dura mater, also dark-coloured, was distended by the contents of the skull; upon dividing it with the scissors there was exposed a brain which had passed in every part into a state of decomposition. Although the line of the convolutions yet remained, it was semifluid, of light green colour, and emitted a most fœtid odour.

Both lungs were emphysematous (from decomposition?)

The heart was healthy, but the muscular structure was soft, and discoloured from the same cause. The pericardium contained about an ounce of turbid serum. The abdominal viscera were soft and rotten; the kidneys, in a similar state, were of large size; the weight of each was twenty-two ounces and a half.

The cancellous texture of the bones was dark from decomposed blood, which had stained the interior in every part not of compact texture. The colour was particularly dark in the cranial bones, the sternum, and the ribs.

